# Epigenetics dysfunction in morbid obesity with or without obstructive sleep apnoea: the EPIMOOSA study

**DOI:** 10.1186/s12931-020-1302-9

**Published:** 2020-02-04

**Authors:** Javier Lázaro, Paloma Clavería, Carmen Cabrejas, José Fernando, Berta Daga, Beatriz Ordoñez, Silvia Segura, David Sanz - Rubio, José M. Marín

**Affiliations:** 1 Respiratory Service, Hospital Royo Villanova, Avda San Gregorio, 50015 Zaragoza, Spain; 2 Endocrinology and Nutrition Service, Hospital Clínico Lozano Blesa, Zaragoza, Spain; 3 Bariatric Surgery Unit, Hospital Royo Villanova, Zaragoza, Spain; 4 Cardiology Service, Hospital Royo Villanova, Zaragoza, Spain; 5 Translational Research Unit, Hospital Universitario Miguel Servet, IIS Aragón, Zaragoza, Spain and CIBER Enfermedades Respiratorias, Madrid, Spain; 6 Department of Medicine, University of Zaragoza, Zaragoza, Spain

**Keywords:** Obstructive sleep apnoea, Morbid obesity, Epigenetics, Exosomes, Bariatric surgery, Continuous positive airway pressure

## Abstract

**Background:**

Obstructive sleep apnoea (OSA) and morbid obesity (MO), defined by a body mass index ≥35 kg/m^2^, are two closely related conditions. Recent studies suggest that circulating microRNA (miRNA) plays a potential role in the physiopathology of both conditions. To date, circulating miRNA expression has been studied separately in both conditions, but never jointly. The primary treatment of OSA is continuous positive airway pressure (CPAP), whereas bariatric surgery (BS) is the treatment of choice for MO. We have thus initiated the Epigenetics modification in Morbid Obesity and Obstructive Sleep Apnoea (EPIMOOSA) study (ClinicalTrials.gov identifier: NCT03995836).

**Methods/design:**

EPIMOOSA is a prospective non-interventional cohort study aiming to recruit 45 MO patients who are candidates for BS. Three groups will be formed: MO without OSA, MO with OSA without CPAP and MO with OSA and CPAP. All of them will be followed up in 4 visits: baseline, 6 months prior to BS and 3, 6 and 12 months post-BS. At baseline, OSA status will be assessed by home sleep polygraphy (HSP), and CPAP will be adopted according to national guidelines. A specific standardized questionnaire (including medical conditions and AOS-related symptoms) and anthropometrical examination will be performed at each visit. Blood samples will be obtained at each visit for immediate standard biochemistry, haematology and inflammatory cytokines. For bio-banking, serum, plasma, and circulating exosomes will also be obtained. Twenty-four hours of blood pressure and electrocardiogram (ECG) Holter monitoring will be performed at all visits. A new HSP will be performed at the last visit. Finally, the three groups will be sex- and age- matched with participants in the EPIOSA study, an ongoing study aimed at understanding epigenetic changes in non-obese OSA patients.

**Discussion:**

EPIMOOSA will evaluate changes in circulating miRNA in MO with or without OSA for the first time. In addition, EPIMOOSA will be able to elucidate the influence of OSA in MO patients and how specific and combined treatments alter miRNA expression.

## Background

The prevalence of obesity, defined as a body mass index (BMI) ≥ 30 kg/m^2^, in the adult population of developed countries is greater than 25% and has reached epidemic levels [1]. Obesity increases the risk of death from many causes [2, 3], being an independent risk factor in the development of several chronic diseases (cardiovascular disorders, type 2 diabetes mellitus [DM2], hyperlipidaemia, cancer, high blood pressure, liver disease, etc.) and acute concurrent processes (e.g., accidents, infections) [4, 5].

Obesity is closely related to the development of obstructive sleep apnoea (OSA) [6] and obesity hypoventilation syndrome [7]. OSA is the most common sleep breathing disorder, affecting 20% of men and 8% of women in Spain [8]. This condition is characterized by repeated episodes of partial or total obstruction of the pharynx during sleep. The main clinical implications are sleep fragmentation and chronic intermittent hypoxia (CIH). OSA and obesity are both associated with high morbi-mortality rates, although the specific role of each condition in a given patient remains unclear.

**Fig. 1:**
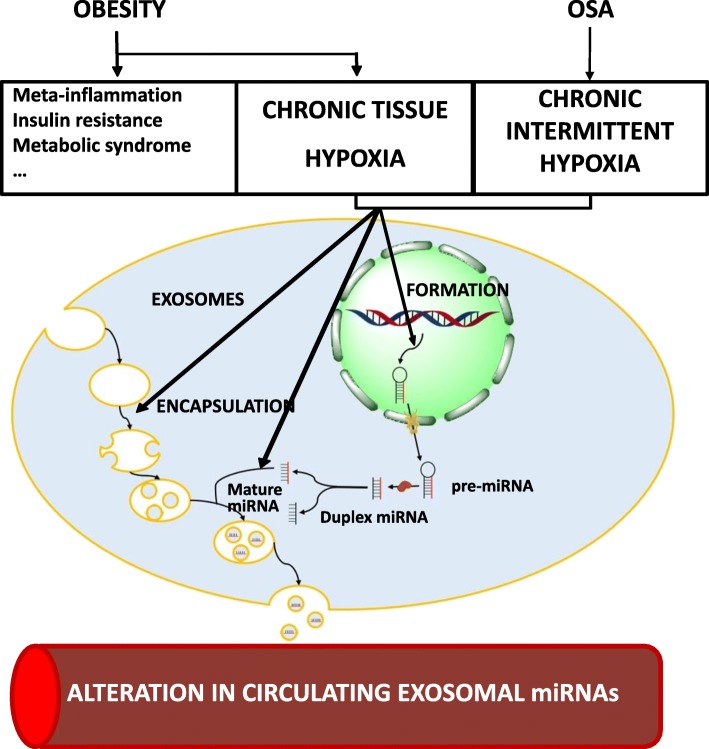
Hypothetical relationship between chronic tissue hypoxia due to obesity and chronic intermittent hypoxia die to obstructive sleep apnoea and the overlap effect on the overexpression of miRNA in circulating exosomes

Adipose tissue from obese patients, regardless of the coexistence of OSA, suffers chronic hypoxia due to its poor vascularization, which increases the level of hypoxia-inducible factor (HIF) [9, 10]. Recent studies have suggested that HIF, which is produced in hypoxic situations, activates different epigenetic mechanisms [11]. OSA has also been associated with the presence of systemic inflammation of as-yet unknown origin. This state may be influenced by epigenetic modifications induced by de-oxygenation/re-oxygenation phenomena, in which CIH is crucial [12, 13]. Both morbid obesity (MO) and OSA patients have an increase in several factors associated with endothelial damage triggered by CIH [14]. Epigenetic changes, especially overexpression of certain miRNAs that target the vascular endothelium (Fig. 1), are an intermediary mechanism that may connect CIH with endothelial damage. In OSA patients without MO, our group has identified an increased level of certain miRNAs in circulating exosomes associated with accelerated atherosclerosis and cardiovascular risk [15, 16]. Interestingly, some of these miRNAs reduced their expression after treatment with nocturnal continuous positive airway pressure (CPAP) [15]. In obese children with OSA, Khalyfa et al. found higher levels of miRNAs from circulating exosomes involved with endothelial dysfunction. Nevertheless, the effect of OSA therapy and weight loss was not evaluated in this study. In addition, no such studies have been performed in obese adults with OSA.

Circulating exosomes are extra-vesicular vesicles that contain lipids, proteins and miRNAs that contribute to remote cell signalling and communication mechanisms in both physiological [17] and pathological processes [18]. This methodological paper describes the aims and methods of the Epigenetics dysfunction in Morbid Obesity and Obstructive Sleep Apnoea (EPIMOOSA) research project, which hypothesizes that obesity and sleep apnoea pathogenically potentiate each other as independent risk factors for cardiovascular morbi-mortality through overexpression of dysregulated miRNAs in circulating exosomes.

## Methods

### Study hypothesis and aim

The hypothesis of the EPIMOOSA study is that patients with a morbid obesity (MO) and OSA have a specific inflammatory phenotype and that, hypoxia-induced epigenetic changes act in synergy with the subclinical state of inflammation inherent to morbid obesity to develop this phenotype. If this hypothesis holds to be true, these changes should be reversed with CPAP and bariatric surgery (BS). In addition, we further aimed to identify epigenetic changes among exosomal miRNAs in MO patients with and without OSA and to determine whether CPAP and BS modify their levels.

### Study design and setting

This is a 2-year prospective, longitudinal, non-interventional cohort study. It will be conducted in the Sleep and Bariatric Surgery Units at the Royo Villanova and Miguel Servet University hospitals in Zaragoza, Spain. The researchers of the EPIMOOSA study have a variety of expertise related to the management of sleep and respiratory disorders, obesity, endocrinology, biostatistics, biomedical informatics, molecular biology and epigenetics. Two scientific centres will provide core services for the analysis of inflammatory biomarkers and genetics (Aragon Health Research Institute, Zaragoza, Spain) and epigenetic data analysis (University of Missouri, Columbia, MO).

### Participant selection and follow-up

Patients will be selected from the bariatric surgery waiting list from both hospitals according to the selection criteria set out in Table 1. At the first visit (baseline), which will include a home sleep study (HSS), the pulmonologist will decide whether to begin CPAP therapy in accordance with Spanish standards [19]. Otherwise, patients will continue to receive their usual care by their primary physician and other specialist throughout the study. All participants will be asked to attend to a 6-month follow-up visit in which adherence to CPAP therapy (if applicable) will be assessed and a date for bariatric surgery will be set if it is still recommended. After BS, patients will attend 3-, 6- and 12-month postoperative follow-up visits.

**Table 1:** Selection criteria

INCLUSION CRITERIA	EXCLUSION CRITERIA
• Age 18–60 years • BMI consistently > 40 kg/m^2^ for 3–5 years, following more than 1 year of unsuccessful controlled medical treatment • BMI 35–40 kg/m^2^ with comorbidities susceptible to improvement with weight loss (hypertension, diabetes mellitus, dyslipidemia, OSA, etc.), following more than 1 year of unsuccessful controlled medical treatment • Signed informed consent form	• Obesity hypoventilation syndrome or treatment with positive pressure devices • Diagnosis of systemic inflammatory disease • Neoplastic diseases in the last 5 years • Previous cardiovascular event (myocardial infarction, stroke or arterial aneurism) in the last 6 months • Pregnancy

The control group will comprise subjects without comorbidities from the EPIOSA study that is currently underway [20]. Patients with and without OSA, adjusted for sex and age (± 2 years), will be selected from the EPIOSA study population, which excludes individuals with a BMI > 35 kg/m^2^.

### Sample size

Three groups of patients will be studied in the EPIMOOSA cohort: a) subjects with MO and without OSA; b) patients with MO and OSA who do not need CPAP therapy; and c) patients with MO and OSA who require CPAP therapy. Each group will include at least 12 subjects who complete the 2-year follow-up after their inclusion at the baseline visit. The sample size was calculated to reveal significant differences in the epigenetic changes between the groups with OSA and the control group (main objective). In a previous study, Kim et al. [21] demonstrated significant differences in the DNA methylation of pro-inflammatory genes among two subgroups of eight children with OSA with high- or low c-reactive protein. In the EPIOSA cohort, we observed significant differences in exosomal miRNA between groups of 12 OSA patients versus controls [16]. Therefore, considering a potential loss to follow-up of 10%, we proposed the inclusion of 15 subjects per group as analysable after a minimum 2-year follow-up, which equates to a final sample size of 45 patients. These 45 patients will subsequently be compared with another 45 cases from the EPIOSA study: 15 subjects without OSA, 15 patients with OSA who refused any treatment and 15 patients with OSA who were treated with CPAP. Therefore, 90 patients in total will be included: 45 from the EPIMOOSA study and 45 from the EPIOSA study.

### Measurements

Table 2 shows the variables that will be collected at each visit.

**Table 2:** Procedures and schedule at EPIMOOSA study

VARIABLE	V0	V1	SURGERY	V2	V3	V4
Time	Baseline	6 m	12 m	15 m	18 m	24 m
Clinical History	*	*	-	*	*	*
Anthropometry	*	*	-	*	*	*
HSP	*	-	-	-	-	*
Blood test	*	*	-	*	*	*
Arterial Blood Gas	*	-	-	-	-	*
miRNA	*	*	-	-	-	*
EKG Holter	*	*	-	*	*	*
24h Blood pressure	*	*	-	*	*	*

*HSP* Home sleep polygraphy. * Done. - Not done

#### Clinical data

The following clinical data and complementary tests will be recorded at each visit: a) sociodemographic data, clinical, surgical and family history, and regular medications; b) level of daytime sleepiness based on the Epworth scale [22]; c) level of depression and anxiety based on the Hospital Anxiety and Depression Scale [23] and the Goldberg scale [24], both in their Spanish versions [25, 26]; d) weight (kg), height (cm), body mass index (BMI = weight (kg)/height (m)^2^), and neck, waist, and hip circumferences (cm); e) blood pressure, measured according to the European Society of Hypertension/European Society of Cardiology clinical practice guidelines [27]; and f) spirometry, measured according to the European Respiratory Society (ERS) standards [28].

#### Home sleep studies

We will use a validated home polygraphy system (ApneaLink Air, ResMed®. Sydney, Australia). The device continuously records airflow, chest movement, oxygen saturation, snoring, and body position. We define apnoea as a lack of airflow for more than 10 s and hypopnea as the reduction of airflow (> 50%) for over 10 s accompanied by a decrease in oxygen levels of more than 4%. The apnoea–hypopnoea index [29] is calculated as the sum of the episodes of apnoea and hypopnoea per hour of recorded time. The results from all sleep studies will be analysed by trained technicians who are blind to the present protocol. Patients will receive CPAP therapy or not based on the recommendations from the Spanish Society of Pulmonology and Thoracic Surgery. This decision will be made by the principal investigator on a patient-by-patient basis according to the severity of AOS, cardiovascular comorbidity, and/or daytime symptomatology. Optimal titration of CPAP will be obtained using auto-CPAP (Autoset-T; ResMed, Sydney, Australia), according to previous validation procedures by the Spanish Sleep and Breathing Group [30]. Compliance with CPAP will be measured using the machines’ internal timers.

#### 24-h blood pressure and electrocardiogram (ECG) Holter monitoring

All patients will complete a 24-h blood pressure and electrocardiogram study. This measure will be performed using a Labtech®*EC*-*3H*/*ABP* (Labtech Ltd., Debrecen, Hungary) that combines a 3-channel *EKG Holter* and an ambulatory *blood pressure* monitor. These studies will be conducted the day after the home sleep study and over the 24-h period before the blood sample collection. They will be interpreted according to the guidelines published by the Spanish Society of Cardiology [31].

#### Blood tests

Fasting blood samples will be collected at each visit. Venous blood samples will be obtained with 21G Abbocaths, and arterial samples will be obtained with 23G ProVent® kits for blood gas analysis. Glucose, triglycerides, total cholesterol, HDL, LDL, and apolipoprotein A and B blood levels will be analysed by spectrophotometry (IMMAGE® 800 Protein Chemistry Analyzer, Beckman Coulter). High-sensitivity C-reactive protein (hsCRP) will be determined within 2 h of collecting the blood samples using flow nephelometry. In total, 15 mL of blood will be used to obtain serum and plasma, and 5 mL of EDTA will be used to conduct genetic and epigenetic studies; these samples will be stored in a freezer at − 80 °C until analysis. Arterial blood gas analysis will be performed at baseline and at the last visit.

#### Exosome and miRNA analysis

Circulating exosomes and the encapsulated miRNA will be studied at the Translational Unit of the Miguel Servet Hospital in Zaragoza. Exosomes will be isolated with the miRCURY™Exosome Isolation Kit (Qiagen, Venlo, Netherlands), and miRNA will be obtained using the miRNeasy Serum/Plasma Advanced Kit (Qiagen, Venlo, Netherlands) as previously described [32]. After extracting the RNA samples, they will be reverse transcribed using the miRCURY LNA™ Universal RT miRNA PCR Kit (Qiagen, Venlo, Netherlands). Mature miRNA will then be quantified by real-time quantitative PCR using PCR Master Mix (Qiagen, Venlo, Netherlands). The integrity of the analyses will be checked using the recommended spike-in control as previously described [32]. The results will be expressed following the 2 -ΔΔ threshold cycle (Ct) method [33]. Table 3 shows the miRNAs that will be studied in the EPIMOOSA study, which will be the same as those in the EPIOSA study.

**Table 3:** Panel of miRNA to be studied in the EPIMOOSA protocol

EPIMOOSA miRNA panel		
UniSP2	miR-320a	miR-16-5p
UniSP5	miR-145-5p	miR-126-3p
cel-miR-39	miR-146A-5p	miR-133a-3p
let 7a-5p	miR-223-3p	miR-34a-5p
miR-21-5p	miR-155-5p	

### Schedule

**Fig. 2:**
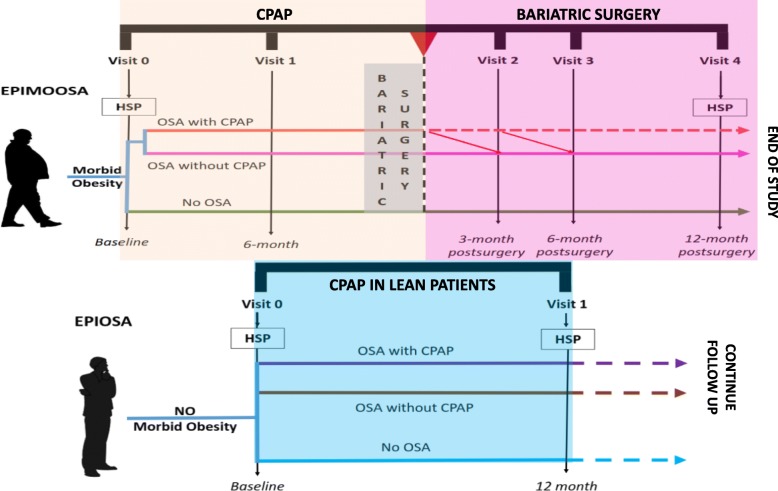
Time – line Follow up. HSP: home sleep polygraphy; OSA: Obstructive Sleep Apnoea; CPAP: Continous Positive Airway Pressure

The study has already been initiated thanks to support from SEPAR and will overlap with the Bariatric Surgery Unit’s care strategy. The recruitment period will last for 2 years, while the follow-up will run for 6 months after the home sleep study and 1 year after surgery. As seen in Fig. 2, we plan to conduct five visits over a 2-year period, excluding the visits associated with bariatric surgery. The study schedule includes two distinct stages. The first stage features patients who have not yet undergone surgery for MO but who are being treated with CPAP for OSA for 6 months, based on their baseline HSP. This period will provide information about the impact of OSA treatment in patients with MO/OSA overlap. The second stage will start after bariatric surgery. This phase involves a 12-month postoperative follow-up with three visits, the last conducted 12 months after the BS because it is at that point when weight loss is thought to be stabilized. As part of the clinical practice at the Sleep Unit, patients on CPAP who have lost at least 20% of their baseline BMI have an additional follow-up home sleep study. If the AHI is lower than 15, the treatment is withdrawn. According to previous experience, most of these patients achieve that goal 3 to 6 months after bariatric surgery. Therefore, to evaluate the effect of BS without the concomitant effect of CPAP, the epigenetic analyses will be performed at least 12 months post-surgery.

### Statistical analysis

We have predefined the following statistical procedures: a) Description of populations. They will be expressed as mean values ± standard deviation for quantitative variables or as numbers and frequencies for qualitative variables. b) Difference between groups: Groupwise comparisons (*n* > 2) will be performed using a nonparametric Kruskal-Wallis test followed by a Mann-Whitney U test when appropriate. Pairwise comparisons were performed using a nonparametric Mann-Whitney test unless otherwise indicated. Chi-square tests will be performed for categorical variables. c) Pearson’s or Spearman’s correlation, depending on the variables’ normality, will be used to evaluate the relationship between systemic inflammation and clinical variables. d) The interindividual variability of miRNA will be determined through the coefficient of variation. GraphPad Prism 8 (GraphPad Software) and SPSS version 23.0 (IBM) statistical packages will be used for all analyses.

## Discussion

Previous studies have evaluated the presence of epigenetic dysfunction in both children and adults with OSA (Table 4). However, most of these studies have assessed epigenetic dysfunction at the DNA methylation level [20, 21, 35, 36] or by using free miRNA in plasma [36, 37]. No such studies have been performed in obese patients with OSA, and no studies have evaluated the effect of CPAP or BS. The epigenetic changes occurring in obesity have also been widely studied, revealing that even perinatal alterations have an influence on the propensity to obesity in adulthood [38]. The authors of a study including 13 obese and non-obese adolescents found 55 different miRNAs (including miRNA 148b, miRNA 4269, miRNA 23b, miRNA 4429) in obese individuals compared to those with a BMI < 25 kg/m^2^, and they were associated with an increase in insulin resistance, which is responsible for target organ damage in obesity [39]. The same group studied the effect of BS-induced weight loss on the exosomes and their content. Another study carried out on six African American women who underwent a gastric bypass examined the mircoRNAs before and 1 year after surgery and reported a change in the expression of 58 miRNAs, 10 of which were directly related to an improvement in insulin resistance [40]. None of these studies have considered the overlap effect of OSA.

**Table 4:** Published works that studied the presence of epigenetic changes in OSA

Study	Population	Primary objective	Epigenetic change	Gene	Patient characteristics
Kim et al. [21]	Children	CRP	DNA methylation	FOXP3	Matched BMI
Khalyfa et al. [34]	Children	ED	miRNA exosomes	–	Obese without OSA vs. nonobese with OSA
Chen et al. [35]	Adults	Severity OSA and EDS	DNA methylation	ILR2, NPR2, AR, SP140	BMI < 35
Kheirandish-Gozal et al. [36]	Children	ED	DNA methylation mRNA	eNOS	Matched BMI
Marin et al. [20]	Adults	Atheromatous plaques in carotid arteries	DNA methylation and expression	FOXp3	BMI < 30
Sanz-Rubio et al. [16]	Adults	Atheromatous plaques in carotid arteries	miRNA exosomes	–	BMI < 30
Sanchez de la Torre et al. [37]	Adults	Reduce BP	miRNA	miRNA 100, 378 and 486	Class 1 obese (BMI 32)

*ED* Endothelial dysfunction, *EDS* Excessive daytime sleepiness, *miRNA* Micro RNA

The adipocytes of patients with MO present hyperplasia, hypertrophy and insufficient angiogenesis, which leads to tissue hypoxia and subsequently negative long-term metabolic and cardiovascular consequences [41]. Hypoxia also causes an increase in cell cytoplasm HIF 1α levels, demonstrating that such an increase activates, among other mechanisms, the release of different miRNAs such as miRNA 21 [42]. Similarly, patients with OSA who endure repeated episodes of nocturnal CIH experience a state of constant hypoxemia that lasts all night and triggers an increase in HIF 1α [43]. These pathophysiological coincidences are limited not only to the HIF but also to endothelial dysfunction in children with OSA and obese children without OSA. These studies revealed that children with endothelial dysfunction exhibited a significant decrease in 4 miRNAs (miRNA 16, miRNA 451, miRNA 5100, and miRNA 630), the last one being of particular interest given its association with endothelial dysfunction [34].

In the context of OSA, different epigenetic markers have been evaluated, specifically DNA methylation and, more recently, the role of miRNAs. Our group has previously studied non-obese (BMI < 30 kg/m^2^) adults with OSA and did not find any changes in methylation or FOXP3 expression. However, we found the dysregulation of certain miRNAs in circulating exosomes (miRNA320-5p and miRNA132-3p) associated with cardiovascular diseases that produced a non-uniform response to CPAP therapy [15]. Those findings explain the increasing interest in this field. On the one hand, epigenetic markers could be useful in identifying subjects at risk of developing certain comorbidities and could be used to monitor the progression of the underlying condition. On the other hand, there are already drugs that act on epigenetic changes, even reversing them and contributing to the treatment of several diseases, especially in the area of oncology [44].

The role of miRNA in OSA is currently a popular area of research. A sub-analysis of the HIPARCO clinical trial [37] revealed that a panel of miRNAs could be used to reliably predict which patients with refractory hypertension and OSA would respond better to CPAP therapy in terms of reducing their blood pressure. An experiment conducted by Khalyfa et al. [45] found a possible pathophysiological relationship among the circulating miRNAs of obese patients with OSA, adipocyte alterations, and the mechanisms of insulin resistance [45].

The relationship between OSA and MO has been well documented [46], showing that their coexistence greatly increases the harmful effects of both conditions on various target organs [47, 48] and that they have intrinsically linked mechanisms of action [43, 49]. Regarding the high prevalence of OSA among morbidly obese patients [7], we believe that the works cited in this article feature a significant methodological bias. The coexistence of two diseases whose close relationship has already been evidenced [50] means that any studies in this area must control both factors carefully.

## Conclusion

Our study aims to evaluate, for the first time, whether patients with MO with/without OSA develop epigenetic dysfunction in circulating exosomes involved in accelerated incidents of cardiovascular diseases. Our study will provide a better understanding of the role of CPAP therapy in patients with MO/OSA overlap and the effect of BS-induced weight loss. The results could also give rise to new biomarkers to improve phenotyping in patients with either or both of the conditions and, possibly, novel therapeutic targets.

## Data Availability

As far as is a study protocol we don’t present any participant information we don’t share any data.

## Abbreviations

BMI: Body Mass Index
BS: Bariatric Surgery
CIH: Chronic Intermitent Hypoxia
CPAP: Continous Positive Airway Surgery
DM2: Type 2 Diabetes mellitus
ECG: Electrocardiogram
ED: Endothelial Dysfunction
EPIMOOSA: Epigenetics dysfunction Morbid Obesity and Obstructive Sleep Apnoea
EPIOSA: Epigenetics modfications and subclinical atherosclerosis in Obstructive Sleep Apnoea
HDL: High Density Lipoprotein
HIF: Hypoxia – inducible factor
HSP: Home Sleep Polygraphy
hsPCR: high sensitive C reactive protein
LDL: Low Density Lipoprotein
miRNA: microRNA
MO: Morbid Obesity
OSA: Obstructive Sleep Apnoea
OSAHS: Obstructive Sleep Apnoea/Hypopnea Syndrome
